# Pilot Randomized Trial Exploring the Impacts of Deceased Donor Kidney Procurement Biopsies on Organ Evaluation and Transplant Outcomes

**DOI:** 10.7759/cureus.81728

**Published:** 2025-04-04

**Authors:** Krista L Lentine, Vidyaratna Fleetwood, Tiffany Caza, Jason R Wellen, Henry B Randall, Richard Rothweiler, Yasar Caliskan, Melissa Lichtenberger, Craig Dedert, Mark A Schnitzler, Huiling Xiao, Yoon Son Ahn, Diane Brockmeier, Gary Marklin

**Affiliations:** 1 SSM Health Saint Louis University Hospital Transplant Center, Saint Louis University School of Medicine, Saint Louis, USA; 2 Nephropathology, Arkana Laboratories, Little Rock, USA; 3 Transplant Surgery, Washington University School of Medicine, Saint Louis, USA; 4 Organ Procurement, Mid-America Transplant, Saint Louis, USA; 5 Clinical Trials Office, Louis University School of Medicine, St. Louis, USA; 6 Scientific Registry of Transplant Recipients, Hennepin Healthcare Research Institute, Minneapolis, USA

**Keywords:** deceased donation, kidney transplantation, organ donor, procurement biopsy, randomized controlled trial

## Abstract

Background: Kidney biopsies obtained at the time of organ procurement are often used in evaluating deceased donor (DD) kidneys. We conducted a pilot randomized controlled trial (RCT) to help assess the feasibility and impact of deferring procurement biopsy information at the time of organ offer.

Methodology: This pilot RCT involved one Midwestern Organ Procurement Organization (OPO) and two local transplant centers in the United States. Waitlisted transplant candidates were approached between July 01, 2019, and May 31, 2022, for consent to receive a kidney from a randomized DD if an offer arose. Criteria for DD randomization to immediate frozen biopsy or permanent section processing (delayed biopsy information) were based on factors used for routine biopsy at the time. Outcomes were assessed through linked registry data.

Results: Of 408 transplant candidates approached, 85 (20.1%) consented to receive a kidney from a randomized DD, if available. Substantial effort was invested in candidate recruitment and consent. Consented candidates disenrolled for reasons including living donor transplantation (9/85, 10.5%), receiving an imported, previously biopsied kidney (11/85, 12.9%), DD outside biopsy criteria (18/85, 21.2%), or immediate frozen biopsy requested (5/85, 5.9%). Eleven randomized DD yielded 12 transplants (6 kidneys from 5 donors in each arm), with a median kidney donor profile index of 49 in the frozen section arm and 54 in the permanent section arm. Biopsy results were similar between arms (median glomerulosclerosis 3.4% vs. 9.9%; *P *= 0.09), with no kidneys exhibiting more than mild interstitial fibrosis or tubular atrophy. Only one biopsy (frozen section arm: 1/7, 14.3%) showed moderate chronic arteriosclerosis. Five frozen biopsies were compared to their post-transplantation permanent section processing, and the median percentage of glomerulosclerosis was similar between reports (1.2% vs.1.2%; *P *= 0.74). None of the frozen biopsies were read as having more severe changes on permanent processing, while two (2/5, 40.0%) showed less severe changes. A single kidney from a randomized donor was unused in the frozen section arm. Unadjusted kidney yield was similar in both groups. Delayed graft function (DGF) after transplant in the frozen vs. permanent processing arms was 50% (3/6) vs. 33.3% (2/6) (*P *> 0.05). Within one year, one recipient from a DD in the frozen section arm died (also with primary non-function), and another experienced graft failure, whereas no events occurred after permanent processing. An RCT designed to detect the observed difference in DGF with power = 0.80 and alpha = 0.05 would require 136 transplants in each arm (272 transplants total). If a similar study achieved the same proportional reduction from consented candidates to randomized transplants, 1,926 consented candidates would be needed.

Conclusions: This pilot RCT did not suggest concerns for delayed DD kidney biopsy information on organ use or transplant outcomes. However, the randomized sample was small, underpowered, and comprised intermediate-risk donors. Trial conduct required considerable effort and faced a high disenrollment rate. Substantial expansion would be required to detect clinically significant differences in outcomes in future trials. Building evidence on the role of kidney procurement biopsies may require national collaboration and consideration of *opt-out* consent models for waiting candidates.

## Introduction

While donor kidney biopsies obtained at the time of organ procurement are often used in decisions to accept or decline kidneys for transplantation, evidence from multiple observational studies suggests that donor kidney biopsies are poor at predicting post-transplant outcomes [1,2]. Donor kidney biopsies, commonly called procurement biopsies, are often obtained under challenging circumstances, including a tissue wedge with subcapsular sampling, utilizing frozen sections that are subject to artifacts, and interpreted by general surgical pathologists without nephropathology training [3]. Perhaps due to these process limitations, the clinical information gleaned from the biopsy frequently correlates weakly with clinical outcomes [4]. A recent survey of U.S. transplant clinicians identified frequent use of the procurement biopsy in organ acceptance decisions, despite a lack of consensus on biopsy indications and adherence to formal criteria, as well as a lack of access to renal pathology specialists or telepathology [5].

Despite these concerns, the number of deceased donor kidneys that undergo biopsy has increased over the past decade [6 ]. The number of nonutilized kidneys has similarly risen. This increase is independent of donor age, comorbidities, cause of death, donor type, and kidney donor profile index (KDPI) but is associated with the use of the kidney biopsy, with nearly one-third of biopsied kidneys discarded [6]. When procurement biopsies are not obtained, non-utilization rates tend to be lower [6-8]. Observational studies have been unable to resolve the debate regarding the appropriate use of procurement biopsies due to selection bias [2], as only organs that were accepted for transplant are included in these analyses. Consequently, these studies cannot truly assess the accuracy of biopsies in assessing the outcome of all recovered organs. In part due to these findings, recommendations of the 2017 National Kidney Foundation (NKF) Consensus Conference to maximize kidney utilization included a statement of a compelling need for a prospective randomized controlled trial (RCT) to establish the benefits and harms of using routine procurement biopsies in decisions for organ acceptance [9].

To explore the ability to prospectively study the impact of frozen section donor biopsy on organ evaluation and transplant outcomes, we performed a pilot RCT involving a Midwestern Organ Procurement Organization (OPO) and two local transplant centers. Eligible deceased donors meeting local criteria for routine biopsy were randomized to immediate frozen-section biopsy versus permanent biopsy processing, such that biopsy results in the permanent section arm were not available to clinical teams during the time of organ acceptance decisions. The goal of this pilot study was to examine the feasibility of performing an RCT to assess whether deferring the release of kidney procurement biopsy information at the time of offer (through delayed permanent biopsy processing) can improve kidney allograft utilization with acceptable recipient outcomes.

## Materials and methods

Study design

This study was a parallel, multi-center pilot RCT conducted in collaboration with the Mid-America Transplant OPO in St. Louis, MO, and two St. Louis transplant centers: SSM Health Saint Louis University Hospital and Washington University/Barnes-Jewish Hospital. The study was approved by the Institutional Review Board (Protocol 29880) and registered under ClinicalTrials.gov Identifier: NCT03837522 (https://clinicaltrials.gov/study/NCT03837522) on February 14, 2019. Waitlist candidates were approached between July 01, 2019, and May 31, 2022. Follow-up for organ offers and transplantation was conducted through June 20, 2022, and outcomes assessment after transplantation ended one year after the last transplant (final assessment November 08, 2022). The conduct of the trial was monitored by an independent Data Safety Monitoring Board (DSMB) using data generated from the Scientific Registry of Transplant Recipients (SRTR).

Study population

The target population of transplant candidates comprised adult patients (age ≥18 years) on the waitlist for kidney transplants at one of the participating hospitals. Candidates for multi-organ transplants and patients unable or unwilling to provide informed consent were excluded. Criteria for routine deceased donor kidney biopsy in the OPO included KDPI > 50%, as well as KDPI components including donor age > 60, Hepatitis C seropositivity, terminal serum creatinine >2.0 mg/dL, history of diabetes, and donation after circulatory death (DCD) status. Donors for whom biopsies were performed *for cause* (e.g., to evaluate a mass of one kidney for cancer or infection) were not included for randomization as a frozen section biopsy was required for disease transmission assessment rather than assessment of allograft quality.

Transplant candidate recruitment

Potential transplant candidate participants were identified by ongoing review of kidney waitlists at participating centers, with a focus on patients considered to be at high likelihood for transplant within 12 months. After screening for eligibility criteria, patients were approached to discuss their interest in participation in the study. The informational brochure used in participant recruitment is included in Appendix A. Participants were contacted either by phone to explain the study and mailed a written copy of the institutional review board (IRB) approved informed consent form for their review or in person at a transplant clinic visit (Appendix A). All patients were informed that their participation was voluntary, that the decision to participate would not affect their care, and that they could withdraw from the study at any time.

Intervention and randomization

**Figure 1:**
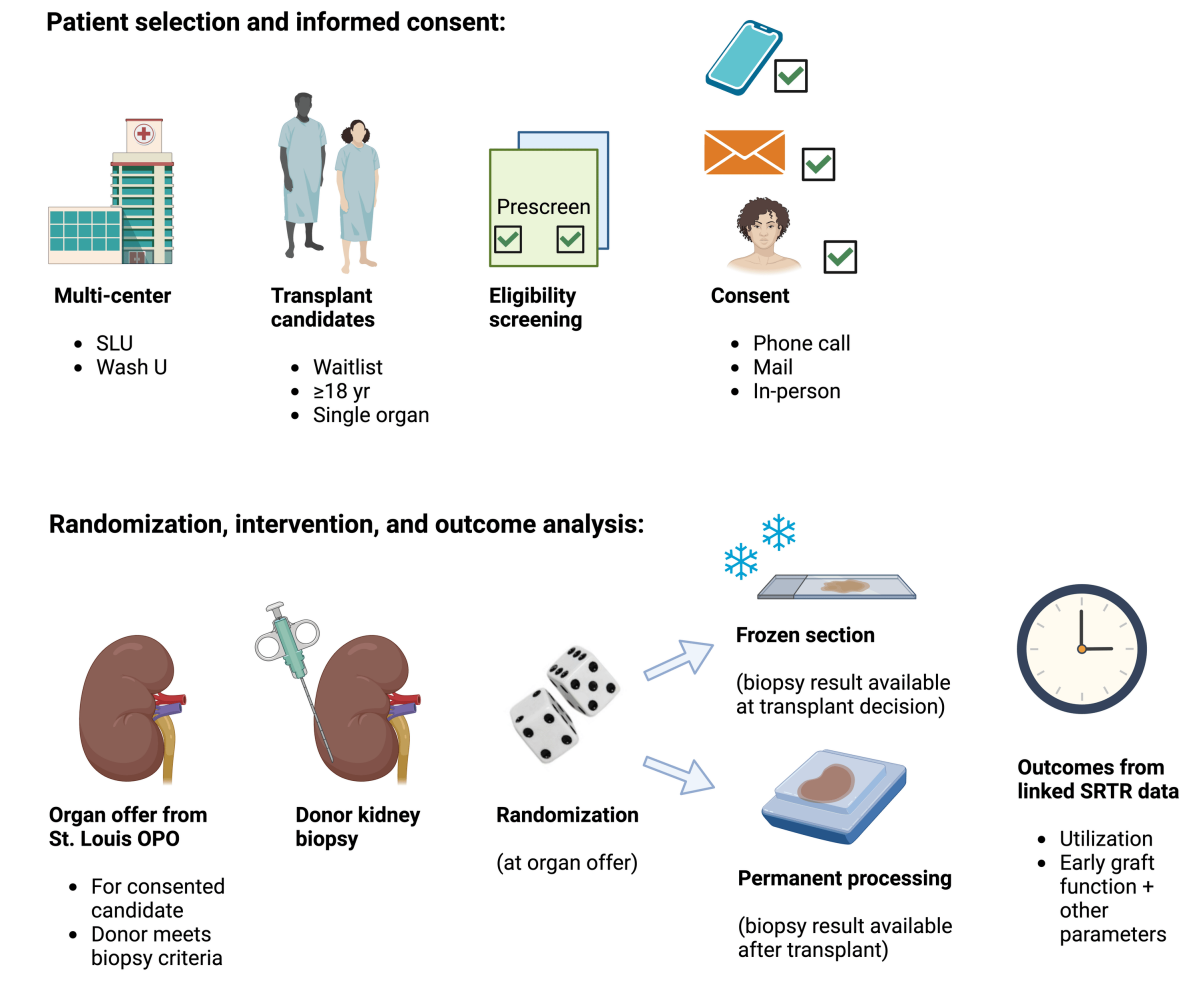
Study design, recruitment, and randomization. (A) Transplant candidates on the waitlist for a deceased donor kidney were identified, and adult patients expected to receive a kidney within a year were contacted for study recruitment. Transplant candidates were contacted by phone or in person during a clinic visit. Consent was performed by mailing consent forms or in person. (B) Consented patients who received an organ offer that met the criteria for kidney biopsy were randomized to the frozen section arm for an immediate biopsy read or to permanent biopsy processing. Outcomes were examined through linkage to SRTR data. SLU, Saint Louis University; OPO, Organ Procurement Organization; Wash U, Washington University in St. Louis; yr, year; SRTR, Scientific Registry of Transplant Recipients The original figure was created by the authors using BioRender, with license: https://BioRender.com/o42i500.

Randomization of biopsy processing was performed at the level of the deceased donor (Figure 1). Deceased donors who met the criteria for routine procurement biopsy, and with at least one kidney allocated to a transplant candidate that consented to participate in the study, were randomized by the OPO using a computer-based number randomizer to one of two conditions: (1) immediate biopsy processing as a frozen section and uploaded into DonorNet when available, per usual practice; (2) biopsy processing by permanent section, producing biopsy results after the offer and allocation complete, generally the next day, with the implanting surgeon unable to access the results until after implantation. Frozen section biopsies were also generally accompanied by additional permanent processing. The OPO team determined donor eligibility and randomly allocated donors 1:1 to the intervention arms if the potential transplant recipient was enrolled. The center teams communicated the study participant status of a transplant candidate to the OPO team at the time of crossmatch. Donors with two kidneys allocated to the same center but only one recipient consented were not included due to the inability to blind the clinical team from contralateral biopsy information. The accepting surgeon had the autonomy to request an immediate frozen biopsy and thus decline donor randomization. 

Data collection

Data collected for the study included characteristics of consented transplant candidates, randomized deceased donors, and transplant recipients, as well as outcomes extracted from the SRTR transplant registry. Patient characteristics included age, sex, race, ethnicity, blood type, diagnosis of kidney failure cause at listing, peak panel of reactive antibodies (PRA), and willingness to accept an expanded criteria donor (ECD)/high KDPI kidney.

Donor characteristics included elements of the KDPI score and other deceased donor risk factors, such as age, race, ethnicity, body mass index, estimated glomerular filtration rate (eGFR), hepatitis C virus (HCV) status, cause of death, and donation after circulatory death (DCD). Additional factors included other viral infection serostatus (hepatitis B core antibody status, cytomegalovirus [CMV] seropositivity, Epstein-Barr virus [EBV] seropositivity), smoking, substance use (alcohol, cocaine, other drugs), vasodilator use before recovery, and Public Health Service (PHS) high-risk classification.

Outcome measures

Outcomes measures include organ disposition (transplant vs non-use), kidney yield per the SRTR formula [10], cold ischemia time, delayed graft function (DGF), eGFR at one year, graft failure within one year of transplant, and patient death within one year of transplant. In addition to SRTR data, we tracked measures of feasibility from the transplant center study teams, including transplant candidate consent rates, time and effort spent per consent achieved, rates of donor randomization, reasons for failure to randomize potentially eligible donors and other donor exclusions, and transplant candidate disenrollments.

Statistical analysis

Our trial was designed as a feasibility pilot to explore the ability to approach this topic in the context of a trial rather than to definitively identify whether delayed biopsy impacted allograft utilization or outcomes. Characteristics of transplant candidates consented for the RCT versus all other candidates listed at the study centers in the study period were compared with the Fisher’s exact test for categorical variables and the Wilcoxon rank sum for continuous variables. Characteristics of deceased donors and included kidney biopsies for RCT participants were compared using the Wilcoxon rank-sum test for continuous variables and Fisher’s exact test for categorical variables. For kidneys assessed by both frozen section biopsy and permanent section results, agreement was evaluated using the paired Wilcoxon signed-rank test for continuous variables and Kappa testing for categorical variables.

Based on the observed DGF rates in the pilot study, we estimated the number of randomized transplants in a future RCT to detect the observed difference in DGF with power = 0.80 and alpha = 0.05, and used the proportional reduction from consented candidates to randomized transplants to estimate the number of consented candidates required in such a study.

## Results

Study recruitment and logistics**

A total of 2,265 adult patients were on the waitlist as transplant candidates at the two participating centers during the study period. Of these, 408 patients deemed likely to receive an organ offer within one year by center staff were outreached for potential study participation, either by in-person discussion at a clinic visit or attempted contact by telephone. Eighty-five patients (20.8%) consented to the study, 45 (11.0%) declined, and the remaining 278 were either unreachable by phone or did not follow up for study participation after receiving the study information. Thus, 130 (31.8%) either accepted or declined trial participation. Study recruitment required pre-screening for eligibility, phone recruitment, mailing information and consent forms, in-person recruitment and consent, and further administrative work, with a mean of eight hours required per waitlisted patient successfully consented to receive an offer from a randomized deceased donor if such a donor became available (Appendices B, C). 

Characteristics of consented transplant candidates 

The study included a total of 85 consented transplant candidates on the waitlist. The majority of participants were men (46, 54.1%), with a median listing age of 57.0 years. Fifty-one (60%) patients were White individuals, and 34 (40.0%) were Black individuals. Causes of end-stage kidney disease included diabetic kidney disease (31, 36.5%), hypertensive nephrosclerosis (14, 16.5%), glomerulonephritis (11, 12.9%), and cystic kidney disease (18, 21.2%) (Table 1)

**Table 1:** Characteristics of transplant candidates consented for the RCT compared to all waitlist candidates at the study centers. Percentages are column percentages. ^a^*P*-value from Wilcoxon rank-sum test for continuous variables or Fisher’s exact test for categorical variables. ECD, expanded criteria donor; IQR, interquartile range; KDPI, kidney donor profile index; PRA, panel reactive antibody; SD, standard deviation

Candidate characteristics	Study participants (*n* = 85)	Candidates not consented for RCT (*N *= 2,180)	*P*-value^a^
Age at listing (years)			
Median (IQR)	57.0 (15.0)	57.0 (20.0)	0.89
Sex, *n* (%)			0.25
Female	39 (45.9%)	863 (39.6%)	
Male	46 (54.1%)	1,317 (60.4%)	
Race, *n* (%)			0.10
White	51 (60.0%)	1,375 (63.1%)	
Black/African American	34 (40.0%)	724 (33.2%)	
Asian	0 (0%)	67 (3.1%)	
Pacific Islander	0 (0%)	5 (0.2%)	
Native American	0 (0%)	6 (0.3%)	
Multiracial	0 (0%)	3 (0.1%)	
Ethnicity, *n* (%)			0.06
Latino	4 (4.7%)	35 (1.6%)	
Non-Latino	81 (95.3%)	2,145 (98.4%)	
Diagnosis at listing, *n* (%)			0.05
Diabetes	31 (36.5%)	719 (33.0%)	
Hypertension	14 (16.5%)	469 (21.5%)	
Glomerulonephritis	11 (12.9%)	329 (15.1%)	
Cystic	18 (21.2%)	252 (11.6%)	
Other	11 (12.9%)	411 (18.9%)	
Blood type, *n* (%)			0.02
A	29 (34.1%)	727 (33.3%)	
B	16 (18.8%)	333 (15.3%)	
AB	7 (8.2%)	62 (2.8%)	
O	33 (38.8%)	1,058 (48.5%)	
Accept ECD/high KDPI (>85%), *n* (%)			<0.001
Yes	47 (55.3%)	506 (23.2%)	
No	38 (44.7%)	1,674 (76.8%)	
Most current PRA level (%), *n* (%)			0.89
<1	43 (50.6%)	1,175 (53.9%)	
1 to <20	11 (12.9%)	256 (11.7%)	
20 to <80	20 (23.5%)	440 (20.2%)	
80 to <98	7 (8.2%)	170 (7.8%)	
98 to 100	4 (4.7%)	139 (6.4%)	
Time on waitlist at enrollment (years)			
Mean (SD)	2.2 (1.2)		
Median (IQR)	2.1 (1.8)		
Time enrolled in study (days)			
Mean (SD)	321 (279)		
Median (IQR)	228 (417)		

On average, patients were listed for a median of 2.1 years at the time of study enrollment. Reflecting targeted recruitment among those with shorter expected time to transplant, fewer participants were of type O blood group (38.8% vs. 48.5%), and more were of type AB blood group (8.2% vs. 2.8%) compared to non-participants. Study participants had a similar calculated panel reactive antibody (cPRA) to non-participants on the waitlist (<20: 63.8% vs.65.6%). A greater proportion of study participants were willing to accept ECD/high KDPI (>85) kidneys (55.3%), compared to 23.2% of non-consented patients (*P *< 0.001; Table 1).

Reasons for disenrollment

**Figure 2:**
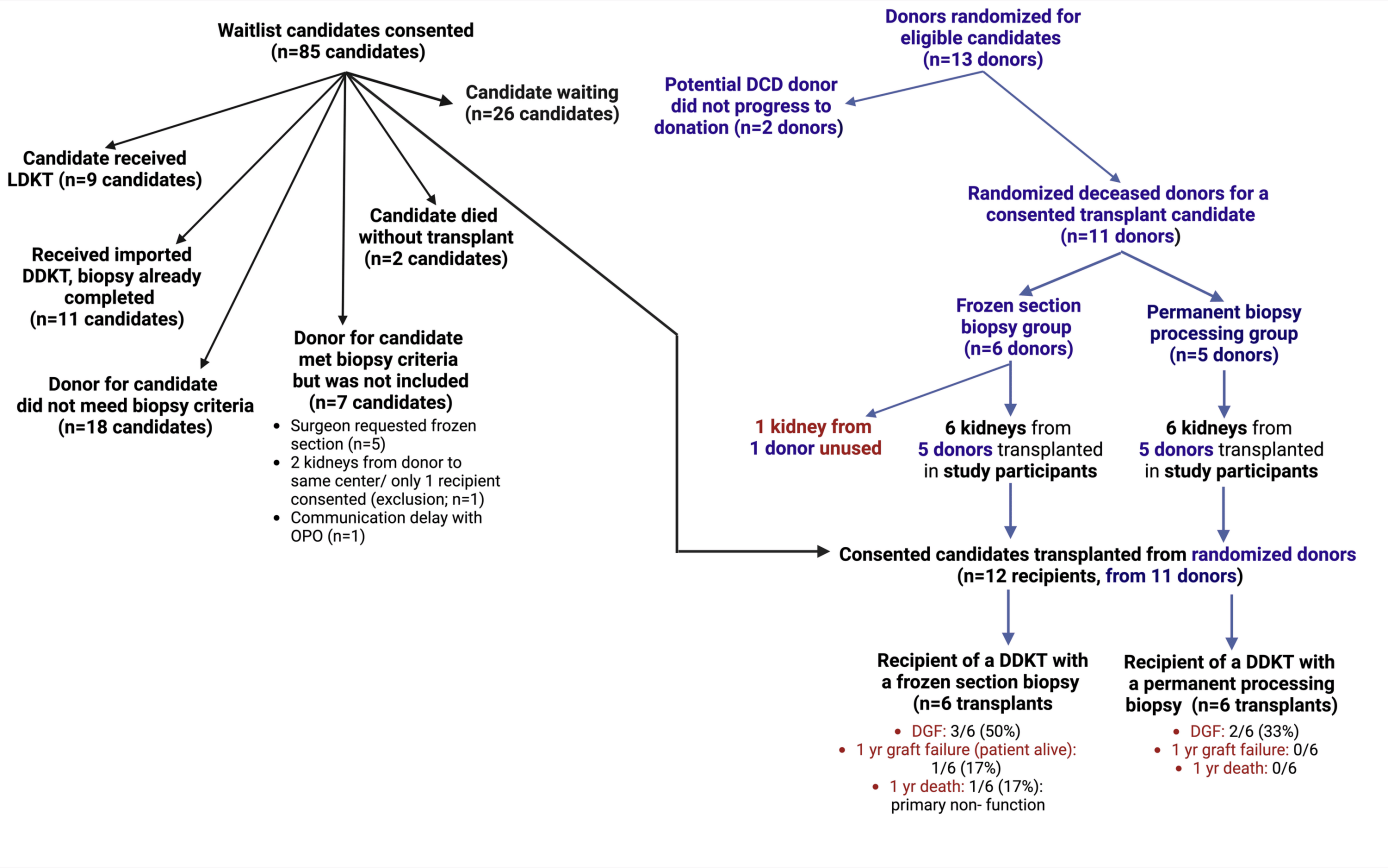
Flowchart of deceased donor allografts and study participant outcomes. Six patients who received randomized kidneys had an immediate frozen section biopsy, and six had biopsies with permanent processing (next-day results, after transplantation). Image credit: All authors. DDKT, deceased donor kidney transplant; DGF, delayed graft function; LDKT, living donor kidney transplant; yr, year

Among the 73 consented waitlisted candidates who did not receive a transplant from a randomized donor (Figure 2), 9 received a living donor kidney transplant and 36 received a deceased donor transplant. Among deceased donor recipients, 11 received a deceased donor kidney imported outside of the OPO with biopsy complete and therefore ineligible for randomization; 18 received a deceased donor kidney transplant from a donor who did not meet biopsy criteria; and 7 received a transplant from a donor who met biopsy criteria but was not randomized (5 due to surgeon request for immediate frozen section biopsy, 1 case of contralateral kidney allocation to a non-consented patient at the same center, and 1 with a communication delay). Two consented candidates died without a transplant during the study period, and 26 were still waiting at the end of the study. Ultimately, 12 study participants were transplanted with randomized donor kidneys.

Outcomes

Study participants were enrolled for a median of 228 days. Thirteen donors who met routine biopsy criteria and had a kidney allocated to a study participant were randomized, including 2 DCD donors who did not progress to donation. Ultimately, 13 kidneys from 11 randomized donors were biopsied. One kidney from one donor in the frozen section arm was not utilized as the list was exhausted without a recipient identified, while 12 kidneys from 10 donors were transplanted in study participants (6 kidneys transplanted from 5 donors in each arm; Figure 2). All kidneys were transplanted as single kidneys and not as dual kidneys. Four kidneys in the frozen section arm were from DCD donors compared to two DCD donors in the permanent section arm (Table 2). The median donor age was 36.5 years in the frozen section group *vs. *50.0 years in the permanent section group (*P *= 0.43). The median donor KDPI score was 49.0 in the frozen section group versus 54.0 in the permanent section group (*P *= 0.36).

**Table 2:** Characteristics of donors by randomized biopsy group (n = 11 donors). Percentages are column percentages. ^a^*P*-value from Wilcoxon rank-sum test for continuous variables or Fisher’s exact test for categorical variables. CMV, cytomegalovirus; DBD, donation after brain death; DCD, donation after cardiac death; IgG, immunoglobulin G; IgM, immunoglobulin M; IQR, interquartile range; KDPI, kidney donor profile index; NAT, nucleic acid testing; PRA, panel reactive antibody; SD, standard deviation

Donor characteristics	Frozen section (*n* = 6 donors)	Permanent processing (*n* = 5 donors)	*P*-value^a^
Age (years)			
Median (IQR)	36.5 (21.2)	50.0 (14.0)	0.43
Sex, *n* (%)			0.55
Female	3 (50.0%)	1 (20.0%)	
Male	3 (50.0%)	4 (80.0%)	
Race, *n* (%)			1.00
White	6 (100.0%)	5 (100.0%)	
Other	0 (0%)	0 (0%)	
Ethnicity, *n* (%)			1.00
Latino	0 (0%)	0 (0%)	
Non-Latino	6 (100.0 %)	5 (100.0%)	
Donor type, *n* (%)			0.57
DBD	2 (33.3%)	3 (60.0%)	
DCD	4 (66.7%)	2 (40.0%)	
Cause of death in DBD			0.60
Anoxia	0 (0%)	2 (66.7%)	
Cerebrovascular/stroke	1 (50.0%)	1 (33.3%)	
Head Trauma	1 (50.0%)	0 (0%)	
CNS Tumor	0 (0%)	0 (0%)	
KDPI			
Median (IQR)	49.0 (26.0)	54.0 (4.0)	0.36
eGFR (mL/min/1.73^2^)			0.90
Mean (SD)	51.6 (11.3)	50.6 (15.4)	
Median (IQR)	45.9 (8.3)	54.7 (23.7)	
Body mass index (kg/m^2^)			
Median (IQR)	24.7 (6.8)	26.4 (2.1)	0.79
History of alcohol use, *n* (%)			1.0
Yes	3 (50.0%)	3 (60.0%)	
No	3 (50.0%)	2 (40.0%)	
History of cigarette use, *n* (%)			1.0
Yes	3 (50.0%)	2 (40.0%)	
No	3 (50.0%)	3 (60.0%)	
History of cocaine use, *n* (%)			1.0
Yes	1 (16.7%)	1 (20.0%)	
No	5 (83.3%)	4 (80.0%)	
History of other drug use, *n* (%)			1.0
Yes	3 (50.0%)	3 (60.0%)	
No	3 (50.0%)	2 (40.0%)	
Vasodilator use, *n* (%)			1.0
Yes	1 (16.7%)	0 (0%)	
No	5 (83.3%)	5 (100.0%)	
Hepatitis C virus, *n* (%)			0.46
Positive	0 (0%)	1 (20.0%)	
Negative	6 (100.0%)	4 (80.0%)	
Hepatitis B virus (NAT), *n* (%)			1.0
Positive	0 (0%)	0 (0%)	
Negative	6 (100.0%)	5 (100.0%)	
Epstein-Barr virus (IgG or IgM), *n* (%)			1.0
Positive	6 (100.0%)	5 (100.0%)	
Negative	0 (0%)	0 (0%)	
Anti-CMV, *n* (%)			0.24
Positive	4 (66.7%)	1 (20.0%)	
Negative	2 (33.3%)	4 (80.0%)	
Public Health Service high risk, *n* (%)			1.0
Yes	2 (33.3%)	2 (40.0%)	
No	4 (66.7%)	3 (60.0%)	

A single kidney from a randomized donor was unused in the frozen section arm, with biopsy findings of 8% global glomerulosclerosis, mild IF, and moderate arteriosclerosis. Unadjusted kidney yield was similar in both groups.

DGF occurred in three recipients transplanted from donors in the frozen section biopsy arm (3/6, 50.0%) and two in the permanent processing biopsy arm (2/6, 33.3%; Figure 2); there was a single case of primary nonfunction (PNF) in a donor from the frozen section arm. Two of the cases with DGF in the frozen section arm were transplanted from DCD donors, while all cases with DGF in the permanent section arm were transplanted from DCD donors. *P*-values for these outcome comparisons were >0.05 due to small samples.

**Table 3:** Characteristics of transplant recipients by donor biopsy randomization group (n = 12 recipients). ^a^*P*-value from Wilcoxon rank-sum test for continuous variables or Fisher’s exact test for categorical variables. IQR, interquartile range; GN, glomerulonephritis; ECD, expanded criteria donor; KDPI, kidney donor profile index; PRA, panel reactive antibody; eGFR, estimated glomerular filtration rate

	Frozen-section biopsy (*N* = 6)	Permanent biopsy processing (*N* = 6)	*P*-value^a^
Age (years) at transplant, *n* (%)			
Median (IQR)	58.0 (14.25)	66.0 (12.75)	0.23
Sex, *n* (%)			1.0
Female	2 (33.3%)	3 (50.0%)	
Male	4 (66.7%)	3 (50.0%)	
Race, *n* (%)			0.58
White	3 (50.0%)	5 (83.3%)	
Black/African American	3 (50.0%)	1 (16.7%)	
Asian	0 (0%)	0 (0%)	
Pacific Islander	0 (0%)	0 (0%)	
Native American	0 (0%)	0 (0%)	
Multiracial	0 (0%)	0 (0%)	
Ethnicity, *n* (%)			1.0
Latino	0 (0%)	0 (0%)	
Non-Latino	6 (100%)	6 (100%)	
Diagnosis at transplant, *n* (%)			0.27
Diabetes	2 (33.3%)	2 (33.3%)	
Hypertension	0 (0%)	2 (33.3%)	
GN	0 (0%)	0 (0%)	
Cystic	2 (33.3%)	2 (33.3%)	
Other	2 (33.3%)	0 (0%)	
Blood type, *n* (%)			0.18
A	3 (50.0%)	5 (83.3%)	
B	0 (0%)	0 (0%)	
AB	3 (50.0%)	0 (0%)	
O	0 (0%)	1 (16.7%)	
Accept ECD/high KDPI (>85), *n* (%)			0.18
Yes	0 (0%)	3 (50.0%)	
No	6 (100%)	3 (50.0%)	
Most current PRA level (%), *n* (%)			1.0
<1%	4 (66.7%)	5 (83.3%)	
1 to <20%	1 (16.7%)	0 (0%)	
20 to <80%	1 (16.7%)	0 (0%)	
80 to <98%	0 (0%)	1 (16.7%)	
98% to 100%	0 (0%)	0 (0%)	
Cold ischemic time (hours)			
Median (IQR)	14.6 (10.7)	19.4 (11.2)	0.40

Kidney function as measured by eGFR at one year was similar in the frozen section and the permanent group, with median eGFR of 45.9 versus 54.7 mL/min/1.73 m^2^, respectively. There was one case of graft failure in a transplant from a donor in the frozen section arm and no graft failures in recipients from donors in the permanent section arm (graft survival: 5/6, 83.3% vs. 6/6, 100.0%]. There was one death in a recipient of a donor in the frozen section arm, with PNF reported as a contributing cause of death, and no deaths in recipients transplanted from donors in the permanent biopsy processing arm (Figure 2).

Biopsy data

Biopsy results were obtained for 13 kidneys from 10 donors, including the non-utilized kidney. All biopsies were taken as wedge biopsies and were deemed satisfactory for evaluation. The mean (5.1% vs. 11.2%; *P *= 0.09) and median (3.4% vs. 9.9%; *P *= 0.09) percentage of glomerulosclerosis was similar between the frozen and permanent section arms (Table 4). Interstitial fibrosis (IF) was present in 5 of 7 (71.4%) biopsies in the frozen section arm and 5 of 6 (83.3%) biopsies in the permanent arm; all IF was categorized as minimal or mild with no moderate or severe changes visualized on biopsy. The majority of biopsies were without significant arteriosclerosis, with only one biopsy with moderate arteriosclerosis (frozen section arm: 1/7, 14.3%). No acute tubular necrosis, fibrin thrombi, or nodular glomerulosclerosis were present in either arm (Table 4).

**Table 4:** Kidney biopsy results according to RCT randomization group (N = 13 biopsies). Percentages are column percentages. ^a^*P*-value from t-test (means) or Wilcoxon rank-sum (median) tests for continuous variables. ^b^*P*-value from Kappa test for proportions. ^*^One kidney from one donor was non-utilized in the frozen section arm. The donor was a 48-year-old White man with a history of smoking and drug use. The cause of death was anoxia/cardiac disease, and the KDPI was 41%. A frozen section biopsy of the donor kidney showed 8% global glomerulosclerosis, mild interstitial fibrosis, and moderate arteriosclerosis. RCT, randomized controlled trial; ATN, acute tubular necrosis

Biopsy results	Frozen section (*n *= 7*)	Permanent processing (*n *= 6)	*P*-value^a^
% glomerulosclerosis, mean (SD)	5.1 (6.8)	11.2 (7.8)	0.09
% glomerulosclerosis, median (IQR)	3.4 (8.0)	9.9 (15)	0.09
	Frozen report (*n* = 7)	Permanent report (*n* = 6)	*P*-value^b^
Interstitial fibrosis, *n* (%)			0.61
Absent	2 (28.6%)	1 (16.7%)	
Minimal/Mild	5 (71.4%)	5 (83.3%)	
Moderate	0 (0%)	0 (0%)	
Vascular changes, *n* (%)			0.50
Absent	2 (28.6%)	1 (16.7%)	
Minimal/Mild	4 (57.1%)	5 (83.3%)	
Moderate	1 (14.3%)	0 (0%)	
ATN, *n* (%)			
No	7 (100.0%)	6 (100.0%)	1.0

Five of the seven biopsies processed via frozen section were also processed as permanent section (Table 5). When comparing these results for paired samples, the mean (5.7% vs. 2.8%; *P *= 0.37) and median (1.2% vs. 1.2%; *P *= 0.74) percentage of glomerulosclerosis were similar between the frozen and permanent section reports. 

**Table 5:** Comparison of kidney biopsy results for kidneys processed as both immediate frozen and permanent sections (N = 5*). ^*^Of the seven biopsies processed through frozen section, permanent processing reports were available for five. ^a^*P*-value from paired t-test (means) or Wilcoxon signed-rank test (medians) for paired samples. ^b^*P*-value from Kappa test for paired samples. ATN, acute tubular necrosis

Biopsy results	Frozen section (*n* = 5)	Permanent processing (*n* = 5)	*P*-value^a^
% glomerulosclerosis, mean (SD)	5.7 (8.3)	2.8 (3.5)	0.37
% glomerulosclerosis, median (IQR)	1.2 (8)	1.2 (4.8)	0.74
	Frozen report (*n* = 5)	Permanent report (*n *= 5)	*P*-value^b^
Interstitial fibrosis, *n* (%)			0.14
Absent	2 (40.0%)	3 (60.0%)	
Minimal/Mild	3 (60.0%)	2 (40.0%)	
Moderate	0 (0%)	0 (0%)	
Vascular changes, *n* (%)			0.02
Absent	2 (40.0%)	3 (60.0%)	
Minimal/Mild	2 (40.0%)	1 (20.0%)	
Moderate	1 (20.0%)	1 (20.0%)	
ATN, *n* (%)			
No	5 (100.0%)	5 (100.0%)	1.0

Inferences for sample size requirements for larger trials 

An RCT designed to detect the observed difference in DGF with power = 0.80 and alpha = 0.05 would require 136 transplants in each arm (272 transplants total). If such a study achieved the same proportional reduction from candidates to randomized transplants, 1,926 consented candidates would be required.

## Discussion

Mounting evidence shows that the kidney procurement biopsy is subject to processing limitations, high interobserver variability,weak reproducibility,and poor association with clinical outcomes [11-14]. However, these biopsies are commonly used, with a recent national survey of U.S. transplant centers showing that over one-third of programs request a biopsy on the majority of kidneys evaluated for implantation [5].Furthermore, the findings on biopsy are frequently given precedence over the clinical markers in decision making, as almost half of the respondents indicated that they would decline a kidney from an otherwise standard criteria donor if biopsy findings were concerning [5]. Given the association between biopsy use with non-utilization rates, the time is right for clinical trials to evaluate both the clinical value of the procurement biopsy and the effect of biopsy blinding on organ evaluation and outcomes. 

Our study sought to assess the feasibility of RCTs to address these questions. Several lessons can be drawn from this pilot study, the foremost being the significant challenge of obtaining informed consent from recipient candidates receiving offers from eligible donors. Despite the frequent ongoing contact of transplant candidates with their waitlisting center, only 31.8% of patients could be reached to discuss the study. The patients who were successfully reached and consented were more likely to be willing to accept ECD/high KDPI kidneys for transplantation. Addressing transplant recipients who were willing to receive high KDPI or increased risk donors may be beneficial in study recruitment as these patients may be less risk averse and willing to accept a kidney without immediate allograft biopsy assessment, especially in the context of equipoise regarding the ability of biopsies to inform appropriate organ use, weighed against the prospect of maximizing the use of all kidneys available for transplant.

Ultimately, only 20.8% of identified patients could be consented. Substantial administrative support was required, including dedicated support from study coordinators. For each patient who consented, an estimated eight hours of administrative work were performed, mostly in prescreening and phone or in-person recruitment. With only 14% of patients receiving transplants from randomized donors, this corresponds to 57 hours per randomized transplant. Efforts focused on reducing administrative work - possibly by exchanging in-person discussion for prerecorded audiovisual content, greater provider involvement in patient explanations and reassurance before coordinator contact, and using electronic methods to obtain consent to reduce administrative burden - may be beneficial to translating this study to a larger trial. Substantial interest has developed, particularly during the time of increased contact limitations and decreased staffing during the COVID-19 pandemic, in reducing the administrative burden in clinical trials. The Biomedical Alliance in Europe (BioMed Alliance, BMA)is a non-profit organization representing 36 European research and medical societies that has published a letter with a call to action to re-evaluate administrative tasks required in clinical trials [15]; a similar document was produced by the European Society of Hematology (ESH) in 2022 [16]. The issues highlighted in both documents included safety reporting, regulatory challenges, and the limitations of informed consent. In our study, the time involved in obtaining informed consent posed the majority of the effort allocation. Informed consent documents can be challenged by a variety of barriers to effective communication: they are frequently lengthy, requiring high health literacy to understand, and are often difficult to translate into other languages [17].Both the BMA and ESH documents recommended reducing the overall length of informed consent documentation to focus mainly on the key points rather than requiring subjects to read and understand full study details. Increased involvement of patient stakeholders was recommended to determine the points most important to the study participants.

Given the feasibility constraints of consenting and maintaining a candidate population to receive offers from selected deceased donors in this context, the transplant community may decide that *opt-out* rather than *opt-in* consent is ethically appropriate in this context. Of note, while our study was not powered for efficacy, there was no trend for increased adverse outcomes with delayed biopsy information, although the sample was intermediate risk. The use of the opt-out consent method has been explored in medical research. A meta-analysis of 15 opt-in versus opt-out study designs noted a generally higher consent rate in opt-out studies (96.8% vs. 84.0%) [18]. Notably, a single study was designed with both opt-in and opt-out arms: consent was 95.6% in the opt-out arm and 21.0% in the opt-in arm. More importantly, the opt-out study design was associated with a more generalizable study population. Studies with opt-in designs were more likely to have subjects who were male and had a higher level of education, higher income, and higher socioeconomic status. Although an opt-out study design is more favorable from the perspective of recruitment and generalizability, it is likely only appropriate for low-risk studies.

Even after waitlist candidate recruitment and enrollment, the transplantation of enrolled candidates with kidneys from deceased donors meeting criteria for routine biopsy was a challenge. Disenrollments were common due to an array of reasons, including receipt of a living donor transplant, transplantation from donors imported outside the OPO, transplantation from a donor who did not meet routine biopsy criteria, and failure to randomize potentially eligible donors due to reasons including surgeon autonomy to request an immediate frozen biopsy. The use of imported kidneys, shipped from a non-local OPO often without an established relationship with accepting physicians, has increased markedly following the recent changes in organ allocation, including the dissolution of local donation service areas and adoption of broader organ sharing circles in March 2021 during the conduct of this trial [19]. A separate publication of local experience demonstrated that the use of imported kidneys increased from 14% to 60% (p<0.001) after the adoption of broader sharing [20]. Further broadening of organ sharing is being planned in the form of continuous distribution [21],which may post barriers to clinical trials that require communication between OPOs and transplant centers.

Within this study, the implanting surgeon requested a study crossover due to concerns about proceeding without a biopsy in 5 cases. This highlights the need for continued discussion of the clinical utility of the biopsy with accepting physicians. Although physician autonomy in decision-making is critical, it is unclear whether surgeons would have required the biopsy given more comfort with the evidence on uncertain utility of procurement biopsies, although the frequent request for frozen section biopsies mirrors practices reported in a national survey [5].

Although procurement biopsies have been associated with an increased frequency of kidney non-use [22], they may have their place. Frozen section biopsies allow for immediate assessment but are subject to frozen artifacts, often have subcapsular sampling that can overestimate the degree of global glomerulosclerosis and tubulointerstitial fibrosis, and are often not interpreted by kidney pathologists [23]. Conversely, permanently processed biopsies have improved histology, can be more readily sampled by a needle core, can be read by a trained kidney pathologist, and ancillary studies can be performed if required. A permanent section biopsy may provide information of pre-existing pathology in the donor, which can identify potential issues early to reduce post-transplant complications and improve survival. This informs the baseline chronicity of the donor kidney and may guide post-transplant care without impacting organ use. A larger-scale study may inform whether permanent section biopsies alone can be used without increased risk to outcomes in routine practice. Further, if improved preservation techniques decrease time pressure for implantation, increased use of permanent section biopsies to replace more rapid frozen section may be possible to replace frozen sections biopsies.

In the future, another possibility for improving donor organ assessment may be to use technological solutions with artificial intelligence (AI). A limitation of donor biopsies is that these are often read by on-call pathologists without training in kidney pathology, who are more likely to be overly conservative in their assessment [13,24]. An AI-based approach to evaluate biopsies with supervised learning has shown a better correlation between biopsy findings and short to intermediate graft survival [25].In a study evaluating frozen sections from 1,560 kidneys, there was >90% concordance of an AI-based read with a trained kidney pathologist. When this kidney donor quality assessment was applied to 398 non-used kidneys, it was predicted that 110 would have similar survival to those transplanted based on biopsy findings, which has the potential to reduce the number of non-use events through more accurate assessment [26]. 

This study has limitations, chiefly related to small and intermediate risk sample, although the pilot study was not designed to detect statistically significant differences in outcomes. Randomization was 1:1 and was not able to balance donor traits within the small, randomized groups. Additionally, the kidney allocation method changed to broader sharing during our trial, causing an increased number of imported kidneys and more difficulty in randomizing donors for consented transplant candidates. However, this pilot trial enabled us to explore feasibility, identify challenges, and inform the development of future trials. To detect the difference in DGF observed in the pilot trial, we estimate a future trial would require 136 transplants per arm (272 in total) and need to consent 1,926 candidates on the waitlist to achieve this number of randomized transplants. Detecting differences in the uncommon events of primary non-function or early graft loss would be challenging and require larger samples. Within the conduct of our study, we identified the need to streamline candidate enrollment in future novel designs, identified the expected frequency of disenrollment due to biopsy or allocation restrictions, and opportunities to educate transplant physicians on the limitations of the procurement biopsy and importance of continued efforts to generate robust evidence related to this practice.

## Conclusions

In conclusion, this pilot RCT did not suggest concerns for delayed deceased donor kidney biopsy information (operationalized through permanent biopsy processing) on organ use or transplant outcomes. We did not observe increases in organ non-use, DGF, graft failure, or death with permanent kidney biopsy processing compared to immediate frozen section biopsy, although the randomized sample was small, relatively low risk, and under-powered for the detection of significant differences in outcomes. Within the small sample, biopsy results were similar in the frozen and permanent section arms, and a comparison of five of the frozen biopsies to their post-transplantation permanent processing showed similar levels of glomerulosclerosis, with no reports of more severe findings on permanent processing.

Trial conduct required significant effort and faced many challenges, including an effort to consent candidates on the waitlist and high disenrollment for reasons including rising organ imports. This study illustrates the considerable effort required to generate evidence on the impact of deceased donor kidney procurement biopsies using a randomized, controlled design with individual transplant candidate consent, suggesting that an RCT powered to detect the observed difference in DGF at a statistically significant level would require 136 transplants in each arm and more than 1,900 consented candidates. Building evidence on the role of kidney procurement biopsies may require national collaboration and consideration of *opt-out* consent models for waiting candidates. Despite the challenges, this work must continue to generate the evidence needed to optimize the use of donated kidneys and to maximize transplant opportunities and outcomes for patients in need of life-saving organ transplants. 

## Figures and Tables

**Figure 3:**
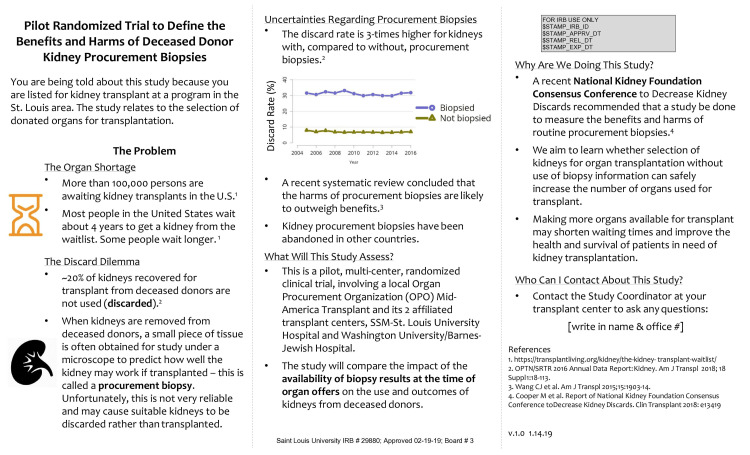
Patient-facing study brochure IRB approved patient-facing study brochure, created by the authors.

**Figure 4:**
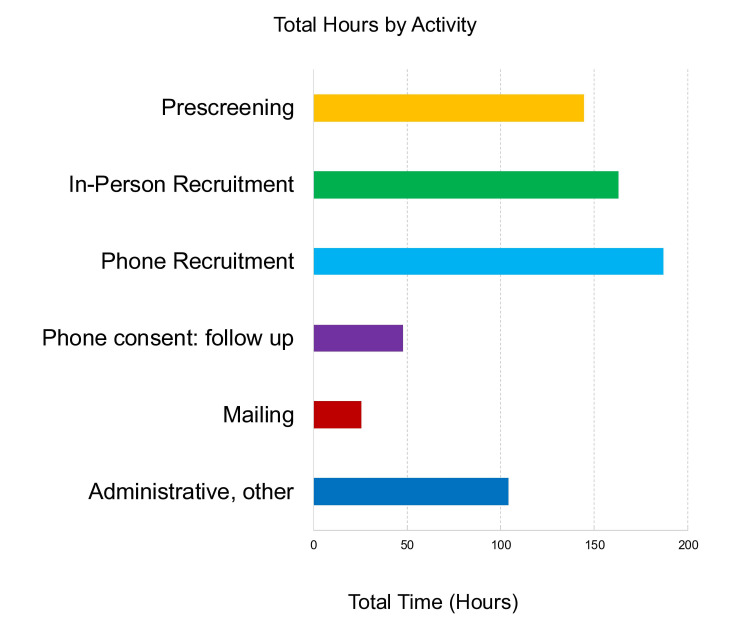
Time spent per activity required to consent potential study participants This included confirmation of transplant candidate status, prescreening for inclusion criteria, phone recruitment, follow-up phone consent, mailing study information and consent forms, in-person recruitment, and additional administrative work. Time spent per activity required to consent potential study participants. This included confirmation of transplant candidate status, prescreening for inclusion criteria, phone recruitment, follow-up phone consent, mailing study information and consent forms, in-person recruitment, and additional administrative work.

**Figure 5:**
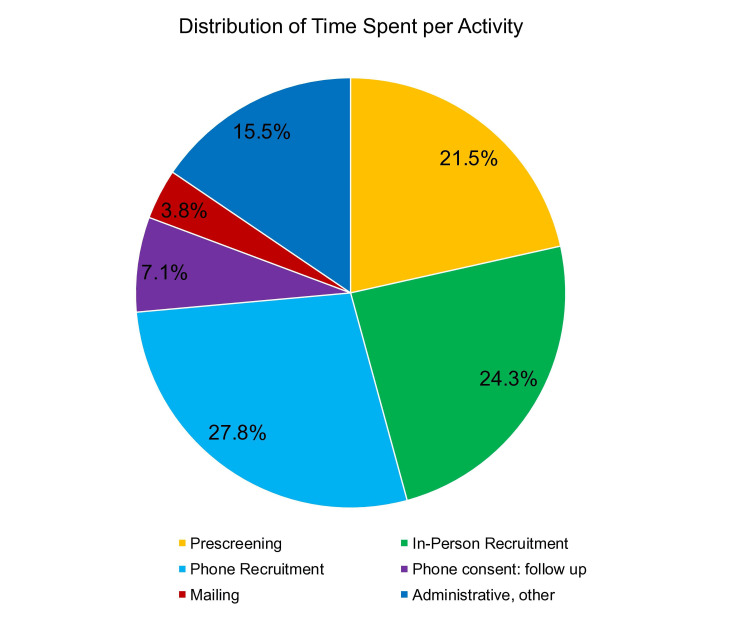
Distribution of time spent per activity.

## References

[1] Wang CJ, Wetmore JB, Crary GS, Kasiske BL (2015). The donor kidney biopsy and its implications in predicting graft outcomes: a systematic review. Am J Transplant.

[2] Lentine KL, Kasiske B, Axelrod DA (2021). Procurement biopsies in kidney transplantation: more information may not lead to better decisions. J Am Soc Nephrol.

[3] Girolami I, Pantanowitz L, Marletta S (2022). Artificial intelligence applications for pre-implantation kidney biopsy pathology practice: a systematic review. J Nephrol.

[4] De Vusser K, Lerut E, Kuypers D (2013). The predictive value of kidney allograft baseline biopsies for long-term graft survival. J Am Soc Nephrol.

[5] Lentine KL, Fleetwood VA, Caliskan Y (2022). Deceased donor procurement biopsy practices, interpretation, and histology-based decision-making: a survey of US kidney transplant centers. Kidney Int Rep.

[6] Lentine KL, Smith JM, Lyden GR (2025). OPTN/SRTR 2023 annual data report: kidney. Am J Transplant.

[7] (2025). Eurotransplant Statistics Report Library. Deceased kidney donors used, by year, by donor country. http://statistics.eurotransplant.org.

[8] (2024). Australia and New Zealand Dialysis and Transplant Registry (ANZDATA). ANZDATA 47th Annual Report 2024 (Data Survey 2023). Chapter 8: Kidney Donation, Deceased Kidney Donors.

[9] Cooper M, Formica R, Friedewald J (2019). Report of National Kidney Foundation consensus conference to decrease kidney discards. Clin Transplant.

[10] Scientific Registry of Transplant Recipients (SRTR (2025). Scientific Registry of Transplant Recipients (SRTR). Measuring donor yield. https://www.srtr.org/about-the-data/guide-to-key-opo-metrics/opoguidearticles/donor-yield/.

[11] Bröcker V, Schubert V, Scheffner I (2012). Arteriolar lesions in renal transplant biopsies: prevalence, progression, and clinical significance. Am J Pathol.

[12] Girolami I, Gambaro G, Ghimenton C (2020). Pre-implantation kidney biopsy: value of the expertise in determining histological score and comparison with the whole organ on a series of discarded kidneys. J Nephrol.

[13] Azancot MA, Moreso F, Salcedo M (2014). The reproducibility and predictive value on outcome of renal biopsies from expanded criteria donors. Kidney Int.

[14] Reese PP, Aubert O, Naesens M (2021). Assessment of the utility of kidney histology as a basis for discarding organs in the United States: a comparison of international transplant practices and outcomes. J Am Soc Nephrol.

[15] Carmona L (2022). Reducing bureaucracy in clinical trials, now is the time!. RMD Open.

[16] Gribben J, Macintyre E, Sonneveld P (2020). Reducing bureaucracy in clinical research: a call for action. Hemasphere.

[17] Kadam RA (2017). Informed consent process: A step further towards making it meaningful!. Perspect Clin Res.

[18] de Man Y, Wieland-Jorna Y, Torensma B, de Wit K, Francke AL, Oosterveld-Vlug MG, Verheij RA (2023). Opt-in and opt-out consent procedures for the reuse of routinely recorded health data in scientific research and their consequences for consent rate and consent bias: systematic review. J Med Internet Res.

[19] Cron DC, Husain SA, Adler JT (2022). The new distance-based kidney allocation system: implications for patients, transplant centers, and organ procurement organizations. Curr Transplant Rep.

[20] Alhamad T, Marklin G, Ji M, Rothweiler R, Chang SH, Wellen J (2022). One-year experience with the new kidney allocation policy at a single center and an OPO in the Midwestern United States. Transpl Int.

[21] (2025). Organ Procurement and Transplantation Network (OPTN). A closer look at continuous distribution of kidney and pancreas. https://optn.transplant.hrsa.gov/policies-bylaws/a-closer-look/continuous-distribution/continuous-distribution-kidney-and-pancreas/.

[22] Lentine KL, Naik AS, Schnitzler MA (2019). Variation in use of procurement biopsies and its implications for discard of deceased donor kidneys recovered for transplantation. Am J Transplant.

[23] Carpenter D, Husain SA, Brennan C (2018). Procurement biopsies in the evaluation of deceased donor kidneys. Clin J Am Soc Nephrol.

[24] Haas M (2014). Donor kidney biopsies: pathology matters, and so does the pathologist. Kidney Int.

[25] Haas M (2024). Improving frozen section evaluation of procurement donor kidney biopsies and reducing the discard rate: a promising role for artificial intelligence. Kidney Int.

[26] Yi Z, Xi C, Menon MC (2024). A large-scale retrospective study enabled deep-learning based pathological assessment of frozen procurement kidney biopsies to predict graft loss and guide organ utilization. Kidney Int.

